# Numerical Study on Effective Conditions for the Induction of Apoptotic Temperatures for Various Tumor Aspect Ratios Using a Single Continuous-Wave Laser in Photothermal Therapy Using Gold Nanorods

**DOI:** 10.3390/cancers11060764

**Published:** 2019-05-31

**Authors:** Moojoong Kim, Gwantaek Kim, Donghyuk Kim, Jaisuk Yoo, Dong-Kwon Kim, Hyunjung Kim

**Affiliations:** Department of Mechanical Engineering, Ajou University, Suwon 16499, Korea

**Keywords:** photothermal therapy, NIR laser, apoptosis, aspect ratio, cancer, hyperthermia, heat transfer, thermal damage, localized surface plasmonic resonance, gold nanoparticles

## Abstract

Photothermal therapy can serve as an alternative to classic surgery in the treatment of patients with cancer. However, using photothermal therapy can result in local overheating and damage to normal tissues. Therefore, it is important to determine effective heating conditions based on heat transfer. In this study, we analyzed laser–tissue interactions in gold nanoparticle (GNP)-enhanced photothermal therapy based on the theory of heat transfer. The thermal behavior inside tissues during photothermal therapy was analyzed using numerical analysis. The apoptosis ratio was defined by deriving the area having a temperature distribution between 43 °C and 50 °C, which is required for inducing apoptosis. Thermal damage, caused by local heating, was defined using the thermal hazard value. Using this approach, we confirmed that apoptosis can be predicted with respect to tumor size (aspect ratio) and heating conditions (laser intensity and radius) in photothermal therapy with a continuous-wave laser. Finally, we determined the effective apoptosis ratio and thermal hazard value of normal tissue according to tumor size and heating conditions, thereby establishing conditions for inducing maximal levels of cell apoptosis with minimal damage to normal tissue. The optimization conditions proposed in this study can be a gentle and effective treatment option for photothermal therapy.

## 1. Introduction

**Figure 1:**
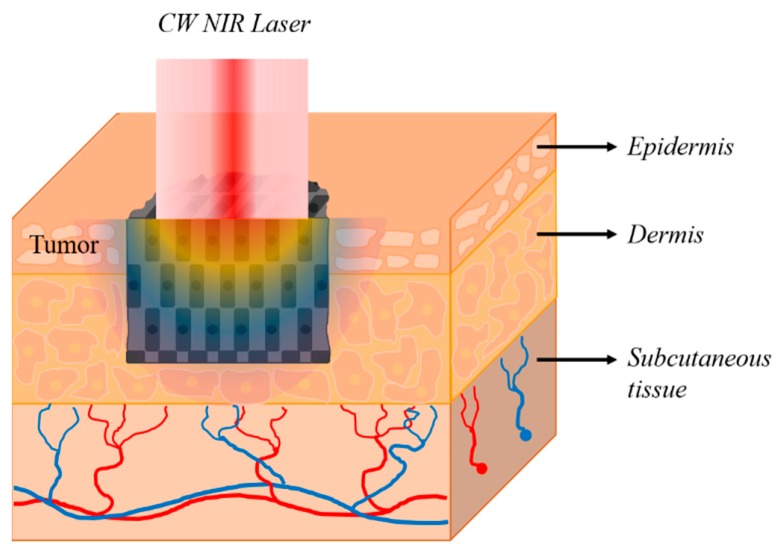
A schematic of photothermal therapy for skin tissue.

Photothermal therapy is a technique for eradicating tumors using the photothermal effect, in which light energy, represented by a laser, is converted to thermal energy as shown in Figure 1 [1]. This new hyperthermic approach has attracted attention as an alternative to classic (conventional) surgery for the treatment of cancer [1,2,3,4]. Photothermal therapy can be performed independently or in combination with other therapies (e.g., radiotherapy and chemotherapy) [5,6,7,8,9].

Thermal energy can be generated by microwaves and electromagnetic waves [10,11]; however, laser-induced thermal treatment (LITT) in the near-infrared (NIR) region is preferable in photothermal therapy because the heating intensity and range in LITT can be easily controlled [12,13]. Biological tissues generally have a very low absorption rate for light in the NIR region. Thus, an optical absorption enhancer is injected into tumors to selectively heat tumor tissues [1,14,15]. Gold nanoparticles (GNPs), such as gold nanospheres (GNSs), gold nanorods (GNRs), gold nanoshells, and gold nanocages, are widely used as optical absorption enhancers in photothermal therapy due to their excellent optical and biological characteristics [6,16,17,18,19].

All cells, including cancer cells, are sensitive to temperature changes and show such symptoms as low activity when the temperature increases; further, excessive heating can lead to cell death [20,21,22]. The death of cancer cells comprising tumors occurs via apoptosis, necrosis, and autophagy [23]. When cell death is caused by necrosis, there is a risk of cancer recurrence and metastasis due to inflammatory response. Therefore, it is important to induce apoptosis. Cell death is induced differently by temperature. Necrosis occurs at temperatures of 50 °C or higher, while apoptosis occurs in the range of 43‒50 °C [20,21,22]. However, even if cancer cells are heated to induce selective apoptosis, it is impossible to evenly heat whole tumors due to thermal diffusion. Thus, because of uneven heating, apoptosis is induced only in a portion of the tumor; the thermal damage associated with this process exerts adverse effects on the surrounding normal tissues [24,25]. Hence, a key objective in photothermal therapy is to minimize the effect of tumor heating on the surrounding normal tissues. This can be accomplished by maintaining appropriate temperatures and inducing apoptosis as uniformly as possible [26].

Hatef et al. [19] used the finite element method to examine photothermal characteristics and temperature changes in a single hollow gold nanoshell dispersed in water using CW (continuous wave), short, and ultrashort lasers. In this previous study, the internal and external temperature distribution changes of a single gold nanoshell were investigated using laser irradiation, and the crystal structure of the gold nanoshell collapsed at 523 K. Ren et al. [27] used periodic heating to investigate the relationships among several factors involved in photothermal therapy. In this study, apoptosis was numerically predicted using the Arrhenius integral, and it was confirmed that the aspect ratio of the tumor was related to optimal apoptosis. Dombrovsky et al. [28] studied the therapeutic effects of asymmetrical periodic heating as compared with those of symmetrical periodic cooling and heating on photothermal therapy. Further, they investigated the temperature change using a numerical model with a geometry similar to that of the actual skin tissue and confirmed that the temperature can be controlled by asymmetric periodic heating. Although these studies provided qualitative data on the properties of heat transfer used in photothermal therapy, they did not quantitatively establish a clear relationship between the rate of apoptosis and the tumor size (represented by the aspect ratio), heating conditions (such as laser intensity and radius), and thermal damage to the surrounding tissues.

In this study, we quantitatively evaluated the apoptosis ratio inside the tumor, as well as the thermal hazard values of the tumor and surrounding tissues, with respect to heating conditions and size alterations of the target tumor (Figure 1). Using this approach, we numerically established the relationships between elements of photothermal therapy, apoptosis, and thermally induced hazard effects on the tumor.

## 2. Theory and Methods

### 2.1. Photothermal Effect

**Figure 2:**
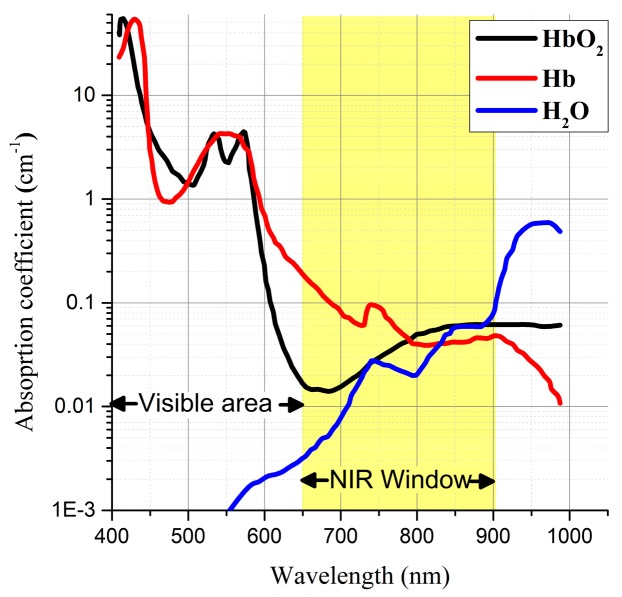
Absorption coefficients of H_2_O, HbO_2_, and Hb with respect to the wavelength [31].

The photothermal effect is a phenomenon in which the energy level of a material increases when photons strike the surface of the material, thereby generating heat. Thus, in terms of heat transfer, photothermal phenomena can be regarded as internal heat generation and can be expressed as a heat source because of the photothermal effect induced by the laser, as shown in the following equation [29]:

(1) $${\dot{q}}_{l}=\mu_{a}\frac{P_{l}}{\pi r_{l}^{2}}e^{-\mu_{tot}\cdot z\cdot \frac{r^{2}}{r_{l}^{2}}}\;(\mu_{tot}=\mu_{a}+\mu_{s}^{\prime })$$

where $\mu_{tot}$ represents the optical properties of the medium consisting of the absorption coefficient ($\mu_{a}$, 1/m) and reduced scattering coefficient ($\mu_{s}^{\prime }$, 1/m); $r_{l}$ is the beam radius of the laser; and $P_{l}$ is the output of the laser. As per Equation (1), even if the laser output is large, the energy of the laser cannot be absorbed if the absorption coefficient ($\mu_{a}$) of the medium is low. The light absorption coefficient of the medium varies with respect to the wavelength of the laser used for irradiation [30]. As shown in Figure 2, HbO_2_ and Hb, present in tumors and normal tissues, possess a high light absorption coefficient in the visible light region. Thus, a light source in the visible light region is inappropriate for selectively heating a tumor. 

For selective heating, we used a laser with a wavelength in the near-infrared (NIR) region. Biological tissues containing H_2_O, HbO_2_, and Hb have a very low rate of optical absorption in the NIR wavelength and can also be optically thin [32]. Because of these properties, photothermal therapy can be used to selectively inject an optical absorption enhancer only into tumor tissues, which have a high optical absorption rate in the NIR region; the tumor tissues can then be subjected to targeted heating with an NIR laser. Gold nanospheres (GNPs) are widely used as enhancers for optical absorption. The wavelength band in which light is not absorbed but passes through biological tissues is called the NIR window. In biological tissues, the NIR window varies depending on the composition ratios of H_2_O, HbO_2_, and Hb, but it is generally between 650 and 950 nm [33,34].

### 2.2. Localized Surface Plasmonic Resonance

**Figure 3:**
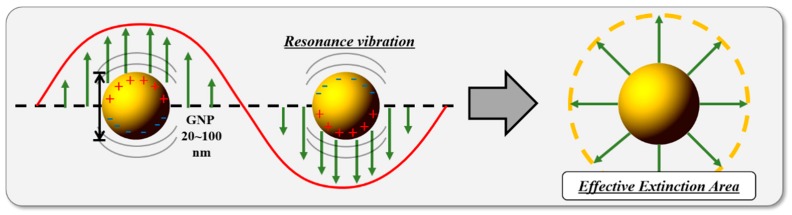
A schematic of localized surface plasmonic resonance.

GNPs generate a large photothermal effect because of localized surface plasmonic resonance (LSPR), which transpires in a specific wavelength band. LSPR is a unique optical characteristic that occurs in nano-sized noble metals. As illustrated in Figure 3, LSPR is a phenomenon in which the surface plasmons (electron group) of nanoparticles, which are smaller than the wavelength of the light source, form a locally increased electromagnetic field by resonating with the wavelength of light, thereby increasing light absorption at a specific wavelength [35]. Thus, plasmon resonance renders the area of light absorption several to several tens of times larger than the actual light absorption area of the nanomaterials, which drastically increases the light absorption rate of a specific wavelength.

The optical properties (absorption ($\mu_{a,\;np}$) and reduced scattering coefficient ($\mu_{s,\;np}^{\prime }$)) of GNPs are determined by the dimensionless efficiency factor (*Q*); *Q,* in turn, is determined by the volume of fraction ($f_{v}$) of GNPs in the medium and the light absorption area increased by LSPR, as shown in the following equation [36]:

(2) $$\mu_{a.np}=0.75f_{v}\frac{Q_{a}}{r_{np.eff}},\;{\mu^{\prime }}_{s,np}=0.75f_{v}\frac{{Q^{\prime }}_{s}}{r_{np.eff}}$$

where the dimensionless efficiency factor varies with respect to the shape and structure of GNPs [37].

Finally, by combining the optical properties of GNPs in the medium with the optical properties of pure medium (biological tissues), the optical properties of biological tissues containing GNPs can be defined as [32,38]:

(3) $$\mu_{a}=\mu_{a.m}+\mu_{a.np},\;{\mu^{\prime }}_{s}={\mu^{\prime }}_{s.m}+{\mu^{\prime }}_{s.np}.$$

### 2.3. Thermally Induced Cell Apoptosis

Existing studies on photothermal therapy estimate the death of cells by using the Arrhenius damage integral as follows [27,28,39]:

(4) $$\Omega (t)=\int_{0}^{t}A_{t}e^{\frac{-E_{a}}{RT(t)}}dt.$$

The Arrhenius damage integral defines thermal damage by the cumulative energy of the medium (biological tissues) over time [40]. Although this is a quantitative index, it only shows permanent thermal damage to the biological tissues. In detail, the permanent thermal damage determined by the Arrhenius damage integral does not indicate biological apoptosis and includes apoptosis, necrosis, biological welding, and carbonization. Moreover, to apply the Arrhenius damage integral, it is essential to observe the transient state of the system; this is inappropriate for applications that enable steady-state analysis, such as photothermal therapy using a CW laser. As mentioned previously, cell death can manifest in various forms, such as apoptosis, necrosis, and autophagy, and the causes of cell death are as diverse as the forms of death [23]. Song et al. [21], Zhu et al. [22], and Ali et al. [20] have shown that apoptosis can be thermally induced by maintaining the temperature of tissues or cells at 43–50 °C. Hence, in our present study, we used a temperature range of 43–50 °C in tumor tissues as our criterion for the induction of apoptosis. The region of the tumor tissues with a temperature of 43–50 °C was defined as the apoptosis volume, and the apoptosis ratio ($\theta_{A}$) was defined as follows:

(5) $$\theta_{A}=\frac{Apoptosis\;volume}{Tumor\;volume}.$$

In other words, the apoptosis ratio proposed in this study is the ratio of the volume within the apoptotic temperature range to the tumor volume. Of course, entering the apoptotic temperature range cannot guarantee that the region immediately undergoes apoptosis. However, the induction of an appropriate range of temperature (represented by the apoptotic temperature range in this study) is a basic requirement for actual photothermal therapy, and quantitative studies on it must be performed for effective treatment.

The apoptosis ratio has a value of 1 when all the tumor tissues undergo induced apoptosis. The apoptosis ratio has a value of 0 if all the tumor tissues fail to reach the temperature criterion for apoptosis or are heated above the criterion temperature range.

### 2.4. Thermally Induced Hazard Effects

According to Jawad et al., various thermal phenomena can occur when biological tissues are heated with a laser, as shown in Table 1 [41]. Apoptosis, which is the object of this study, occurs in the range of 43–50 °C; beyond this range, unintended effects such as protein denaturation, biological welding, and carbonization can occur. When the absorption of the laser (light source) is increased via optical absorption enhancers such as GNPs, thermal damage to the tumor and surrounding tissues is inevitable in photothermal therapy; this occurs because of local overheating (over 50 °C).

In terms of photothermal therapy, unintended biological phenomena resulting from overheating of biological tissues are fatal as the temperature increases. Therefore, the damage caused by thermal effects needs to be quantitatively analyzed with respect to tumor size and heating conditions. For this purpose, as shown in Table 1, weights are given for each temperature range. Here, the weight is a relative indicator of the thermal hazard from the temperature increase. For the purpose of photothermal therapy, tumors have a weight of 1 when the temperature is 37 ≤ T < 43 or 43 ≤ T < 50. This means that the tumors are thermally safe at or below the apoptotic temperature range. A normal tissue has a weight of 1 only if the temperature range is 37 ≤ T < 43; this is because it is not safe for normal tissues to undergo apoptosis or other thermal damage. In this study, we defined the thermal hazard value as $\theta_{H}$, as shown in Equation (6), using the weighted sum of the volume at each temperature range (${V_{i}|}_{T=range}$), and the weight of each range ($w_{j})$ is listed in Table 1 according to the tumor and surrounding tissues. According to Table 1 and Equation (6), a thermal hazard value ($\theta_{H}$) of 1 for the tumor and normal tissues indicates that the tissues have not sustained any damage via thermal effects. Conversely, if rapid cutting and ablation are caused by excessive heating to 300 °C, the thermal hazard value ($\theta_{H}$) is 7 in tumor tissue or 8 in normal tissue.

(6) $$\theta_{H,i}=\frac{\sum_{j=1}^{n}\left({V_{i}|}_{T=range}\cdot w_{j}\right)}{V_{i}}\left(i=\begin{array}{c} t:\;tumor\;tissues\;\\ n:\;normal\;tissues \end{array}\right)$$

Even if the heating conditions are set for the effective apoptosis ratio with respect to tumor size, they cannot be used if thermal damage to the tumor and surrounding tissues is high. Therefore, it is important to derive heating conditions that have an optimal $\theta_{A}$/$\theta_{H}$ ratio with respect to tumor size.

### 2.5. Governing Equation

Photothermal therapy involves heat transfer with internal heat generation in biological tissues. The internal heat generation used in photothermal therapy includes heat generated by metabolism (${\dot{q}}_{mt}$), heat generated by blood flow (${\dot{q}}_{b}$), and heat generated by the photothermal effect (${\dot{q}}_{l}$). This is represented by the Pennes bioheat governing equation as shown in Equation (7) [19,27,28,42]:

(7) $$-k\nabla^{2}T={\dot{q}}_{l}+{\dot{q}}_{mt}+{\dot{q}}_{p}$$

where

(8) $$\begin{array}{cc} \bar{T}=\frac{T-T_{\min}}{T_{\max}-T_{\min}} & ( \end{array}T_{\min}=T_{room})\;,\;{\bar{x}}_{i}=\frac{x_{i}}{L_{c}}\;(i=1,2,3)\;,\;L_{c}=l_{t}.$$

This heat transfer problem can be simplified by using dimensionless temperature ($\bar{T}$) and dimensionless length ($\bar{x}$) in Equation (8) [43,44,45]. In a dimensionless process, the heat generated by metabolism (${\dot{q}}_{mt}$) and that generated by blood flow (${\dot{q}}_{b}$) are very small compared with the heat generated by the photothermal effect; thus, ${\dot{q}}_{mt}$ and ${\dot{q}}_{b}$ were disregarded in this study [46,47]. Equation (7) can be converted to Equation (9) by using dimensionless temperature and dimensionless length. Thus, the dimensionless heat generation (S) changes depending on the aspect ratio ($l_{t}/2r_{t}$) of the tumor and the ratio of the laser–tumor radii ($r_{l}/r_{t}$); the apoptosis ratio of the tumor (ratio of the area having a temperature of 43–50 ℃) is closely related to the $T_{max}-T_{min}$ value of the tumor.

(9) $$\frac{d^{2}\bar{T}}{dx_{1}{}^{2}}+\frac{d^{2}\bar{T}}{dx_{2}{}^{2}}+\frac{d^{2}\bar{T}}{dx_{3}{}^{2}}=-S\;,\;S={\frac{P_{l}}{kL_{c}(T_{\max}-T_{\min})}\;|}_{\begin{array}{l} \mathrm{Aspect}\;\mathrm{ratio},\\ \mathrm{Radius}\;\mathrm{ratio} \end{array}}$$

This fact is important considering the heat transfer phenomenon; this is because regardless of the absolute size of the tumor, if the aspect ratio of the tumor ($l_{t}/2r_{t}$) and laser–tumor radius ratio ($r_{l}/r_{t}$) are identical, the same heat transfer situation appears (e.g., the same temperature distribution, apoptosis ratio, and thermally induced hazard value).

### 2.6. Numerical Analysis

As shown in Figure 1, in this study, the employed cancer model was a skin tumor. Most of the actual tumors, including the skin tumors employed in this study, have a very complex geometry. In addition, tumors have inhomogeneous characteristics due to tissue differentiation and variation between patients. However, numerical analysis considering both the complex geometry and inhomogeneous characteristics of actual tumors is very complex and realistically difficult. This study is performed as the first step of the quantitative analysis of photothermal therapy. For this, it is desirable to select a simplified model compared to the actual tumor.

**Figure 4:**
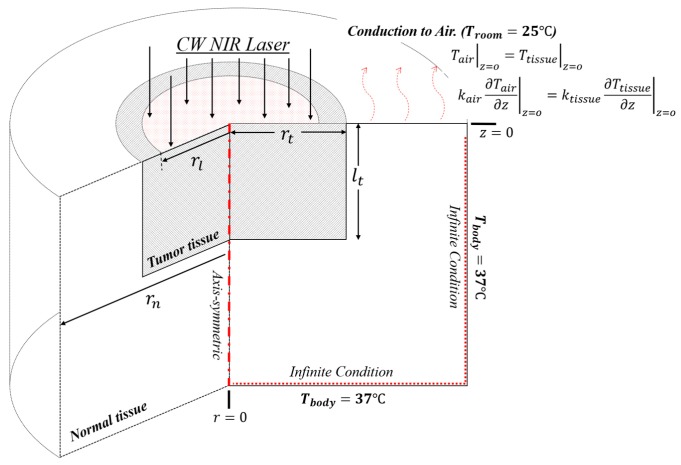
A schematic of the geometry and boundary conditions for numerical analysis.

For a simplified approach to the purpose of this study, numerical analysis using a two-dimensional axisymmetric model was employed as shown in Figure 4. This model was used as described in the numerical heat transfer studies by Ren et al. [27] and Dombrovsky et al. [28]. For verification of the numerical analysis, the numerical model was constructed with the same boundary condition as that in the previous numerical analysis model.

**Figure 5:**
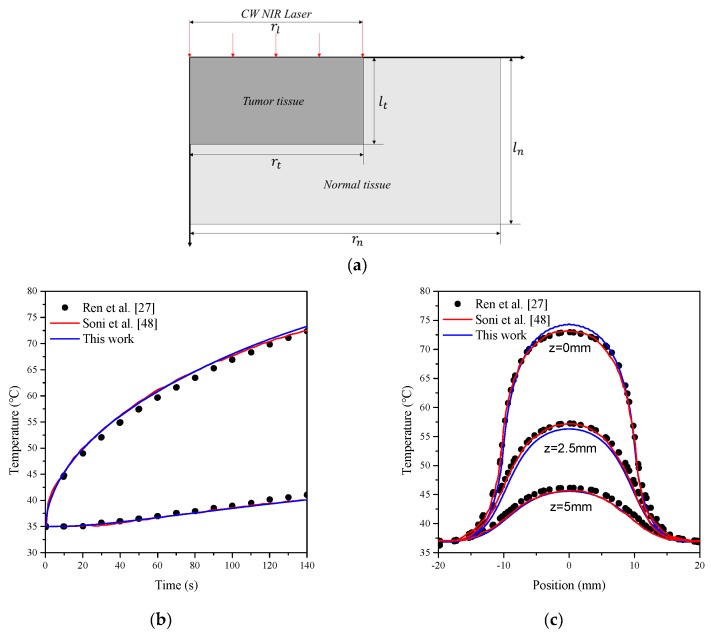
(**a**) A schematic of the geometry and (**b**, **c**) comparison of results for the verification of numerical analysis [27,48].

Figure 5 shows the research results of Ren et el. [27] and Soni et al. [48] and the verification of the numerical analysis of this study. The model schematic is shown in Figure 5a. The geometry dimensions were set as $r_{t}=10\;\mathrm{mm}$, $l_{t}=5\;\mathrm{mm},\;r_{n}=20\;\mathrm{mm}$, $l_{n}=10\;\mathrm{mm},\;\mathrm{and}\;r_{l}=10\;\mathrm{mm}$. The boundary conditions were (1) upper boundary: natural convection with heat transfer coefficient $h=5\;W/\left(m^{2}\cdot K\right)$ and room temperature $T_{room}=25\;℃$; (2) left boundary: symmetry; (3) right and bottom boundaries: $T_{body}=37\;℃$. The initial temperature of tissues was set as 35 °C. In the verification, the incident laser power intensity was 0.5 $W/m^{2}$. The temperature change over time was acquired, and the time step of the numerical model was 0.1 s. The thermal and optical properties used for the verification of the numerical analysis are summarized in Table 2. Then, the modified boundary conditions (e.g., Gaussian laser profile, infinite condition of body temperature, modified heat sources) were applied to the validated model to construct the numerical analysis model employed in this study.

The amount of laser energy absorbed by air was disregarded in this study, because although the laser passes through air during photothermal therapy, the light absorption rate of air is very low [49,50]. The laser was absorbed by tumors containing GNPs and was transferred to the upper air layer at the temperature of 20 °C via conduction. As shown in Figure 4, this evaluation was conducted using infinite conditions under which the body temperature of 37 °C was maintained at the side and bottom of the biological tissues. 

In this numerical analysis model, we evaluated the optical and thermal properties. The optical properties can be divided into those of the skin and those of GNPs. We applied Caucasian skin as the characteristic of tissues in the numerical model and gold nanorods (GNRs) as the GNPs for our analysis. We applied 788 nm as the wavelength of the light source, because this is the wavelength at which GNRs show maximum dimensionless efficiency (Q). According to various studies, the thermal properties of the skin can differ. In this study, we used the values from Ratovoson [51]. The detailed values of the properties applied in the numerical analysis model are outlined in Table 3. The thermal properties of the tumor may change when GNRs are injected, but the volume fraction of GNRs ($f_{v}$) in tumor tissues is very small (10^−5^). Thus, changes in the thermal properties of the GNPs were not considered in this study.

As described in Section 2.5, we only considered the aspect ratio of the tumor ($l_{t}/2r_{t}$). For a tumor radius ($r_{t}$) of 2 mm, the ratio of cell death was examined when the tumor length ($l_{t}$) was changed to 5 mm and the aspect ratio was changed stepwise from 0.1 to 1.25.

**Figure 6:**
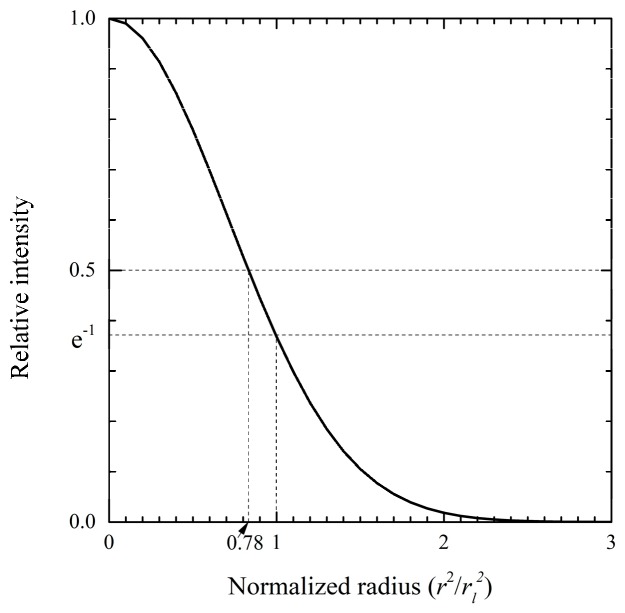
The radius of a Gaussian laser.

The laser intensity ($P_{l}$) was changed from 0 to 0.2 W using 0.005 W intervals for tumors having all aspect ratios. When a CW laser is used, a steady state with no temperature change is assumed for the target biological tissues. Thus, the power of the laser considered in this study had a lower energy than that used in the periodic heating method of previous studies [27,28]. In previous studies on heat transfer in photothermal therapy [27,28], the laser profile was assumed to have a top-hat distribution. However, as shown in Figure 6, the native profile of a laser has a Gaussian distribution. Thus, an additional optical device is required to create a top-hat distribution, and the created distribution is different from the theoretical top-hat profile [52]. For this reason, we used a laser with Gaussian distribution as shown in Equation (1). The radius of a Gaussian laser is the distance from the maximum intensity to 1/e in the distribution; this is indicated by the radius ($r_{l}$) of the laser shown in Equation (1). In this study, the radius was based on the radius of the tumor ($r_{t}$), and $r_{l}/r_{t}$ was evaluated in the range of 0.2‒2.

The unit of the absorption coefficient ($\mu_{a}$) is m^−1^, and it is the reciprocal of the depth to which light can penetrate after accounting for absorption and scattering. 

Based on the optical properties of the GNRs used in this study, the absorption coefficient of the tumor containing GNRs at a volume fraction ($f_{v}$) of 10^−5^ was approximately 34,000 m^−1^; this constitutes a penetration depth of 29 μm, which is very shallow with regard to tumor length ($l_{t}$). Hence, we only used a GNR volume fraction ($f_{v}$) of 10^−5^.

The photothermal therapy parameters evaluated in our numerical analysis were 1 GNR volume fraction ($f_{v}$), 8 laser radii ($r_{l}$), 24 tumor aspect ratios ($l_{t}/2r_{t}$), and 41 laser powers ($P_{l}$). These parameters are described in Table 4.

## 3. Results and Discussion

As described in Section 2.5, the results obtained using our analysis model can be simplified by nondimensionalization. Therefore, our results can be used to find the effective laser intensity for inducing maximal rates of apoptosis in tumors according to the aspect ratio ($l_{t}/2r_{t}$), laser–tumor radius ratio ($r_{l}/r_{t}$), and tumor length (characteristic length, $\;L_{c}=l_{t}$).

**Figure 7:**
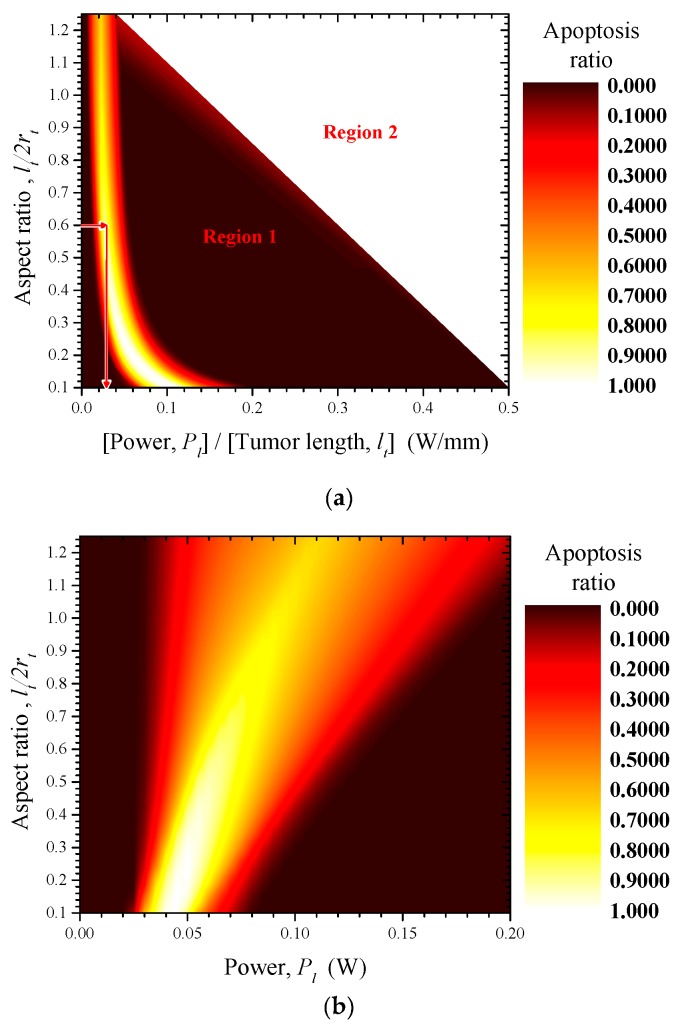
(**a**) The apoptosis ratios ($\theta_{A}$) of tumors according to the aspect ratios of the tumors ($l_{t}/2r_{t}$) and laser power per unit tumor length ($P_{l}/l_{t}$) (radius ratio: 1.00); (**b**) The apoptosis ratios ($\theta_{A}$) of tumors according to the aspect ratios of the tumors ($l_{t}/2r_{t}$) and laser power ($P_{l}$) (radius ratio: 1.00).

Figure 7a shows the apoptosis ratio ($\theta_{A}$) of a tumor when the laser–tumor radius ratio ($r_{l}/r_{t}$) is 1.00. The vertical axis indicates the aspect ratio of the tumor ($l_{t}/2r_{t}$), and the horizontal axis indicates the laser power per unit tumor length ($P_{l}/l_{t}$). For example, when the tumor radius is 5 mm and the tumor length (characteristic length, $\;L_{c}=l_{t}$) is 6 mm, the aspect ratio becomes 0.6; apoptosis is maximized when the laser power per unit tumor length is approximately 0.03 W/mm. Thus, the laser power ($P_{l}$) required to induce maximum apoptosis is approximately 0.18 W.

Due to the setup of the parameters used for numerical analysis, all contour graphs constructed in this study show a trapezoid shape, as does the example shown in Figure 7a. These results were due to the setting ranges of laser power ($P_{l}$) and tumor length ($l_{t}$), used as parameters. The results for the parameters of Table 4 used in this study are shown in Figure 7b. The results from Figure 7b are applicable only to tumors in the ranges shown in Table 4 since they are only relevant for the parameters of this study. As described above, the results of this study can be applicable regardless of the absolute size of the tumor by nondimensionalization using the characteristic length ($L_{c}=l_{t}$) of the tumor. Therefore, Figure 7a, which shows the horizontal axis as the laser power per unit tumor length ($P_{l}/l_{t}$), can clearly explain the biophysical meaning to be emphasized in this study.

This shape of the contour graph did not affect the results of our analysis. We examined the trend of the tumor apoptosis ratio in the analyzed region (Region 1 in Figure 7a) and the unanalyzed region (Region 2 in Figure 7a). The unanalyzed region showed a very low ratio of tumor apoptosis (0–0.3); this was due to the overheating of tumor tissue. Therefore, because the purpose of this study is to optimize the induction of apoptosis via photothermal therapy, Region 2 in Figure 7a was deemed unnecessary.

### 3.1. Apoptosis Ratio and Thermal Hazard Value of Tumor Tissue

**Figure 8:**
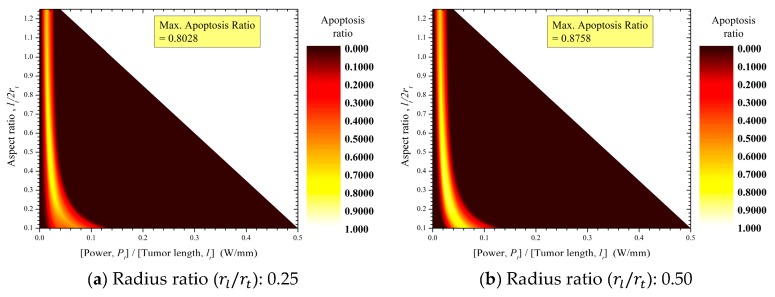

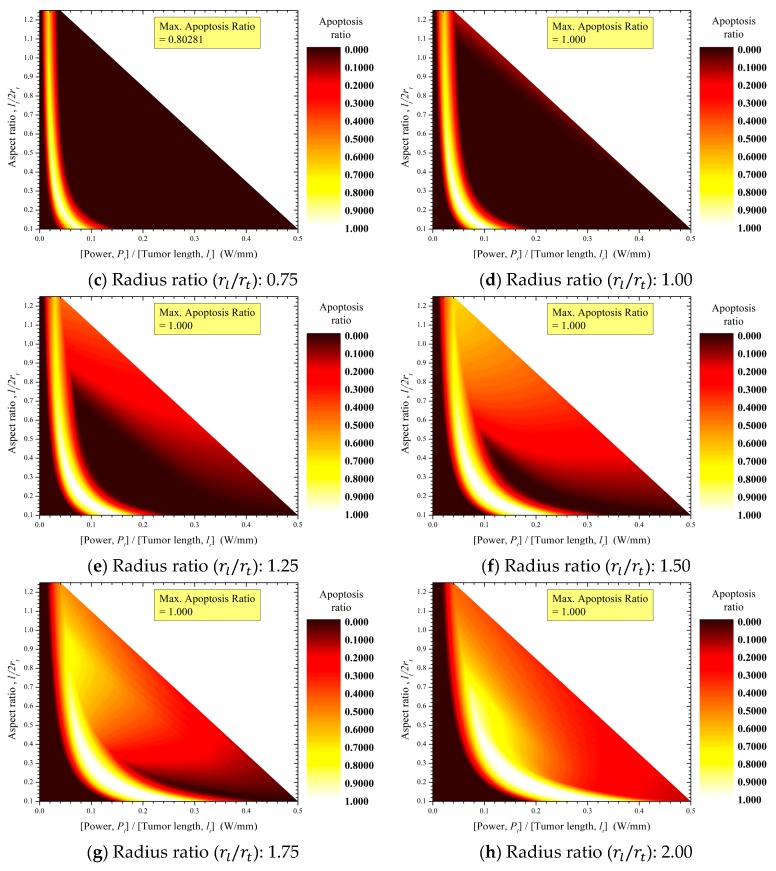
Contour graphs of the apoptosis ratios ($\theta_{A}$) in tumor tissues.

Figure 8 shows the apoptosis ratios ($\theta_{A}$) of tumor tissues according to tumor aspect ratio ($l_{t}/2r_{t}$) and laser power per unit tumor length ($P_{l}/l_{t}$) for various laser–tumor radius ratios ($r_{l}/r_{t}$). At every laser–tumor radius ratio ($r_{l}/r_{t}$), there is a laser power per unit tumor length ($P_{l}/l_{t}$) showing the maximum ratio of apoptosis.

**Figure 9:**
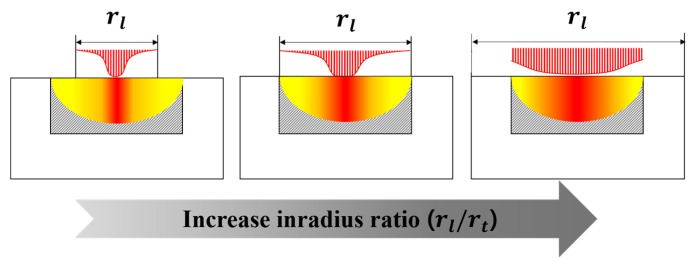
Laser profile changes with increasing radius ratio ($r_{l}/r_{t}$).

Increases in the laser–tumor radius ratio ($r_{l}/r_{t}$) are accompanied by increases in the range of aspect ratio ($l_{t}/2r_{t}$) at which the maximum apoptosis ratio ($\theta_{A,max})$ occurs and by increases in laser power per unit tumor length ($P_{l}/l_{t}$) and its range. This trend is due to the relatively uniform heat that is induced in a thin tumor (in which the aspect ratio ($l_{t}/2r_{t}$) is small) as the laser profile affecting that tumor gradually becomes flat (Figure 9). For the same reason, the maximum apoptosis ratio increased concurrently with increases in laser radius ($r_{l}$), and the maximum apoptosis ratio converged to 1 as the laser–tumor radius ratio ($r_{l}/r_{t}$) exceeded 1.

**Figure 10:**
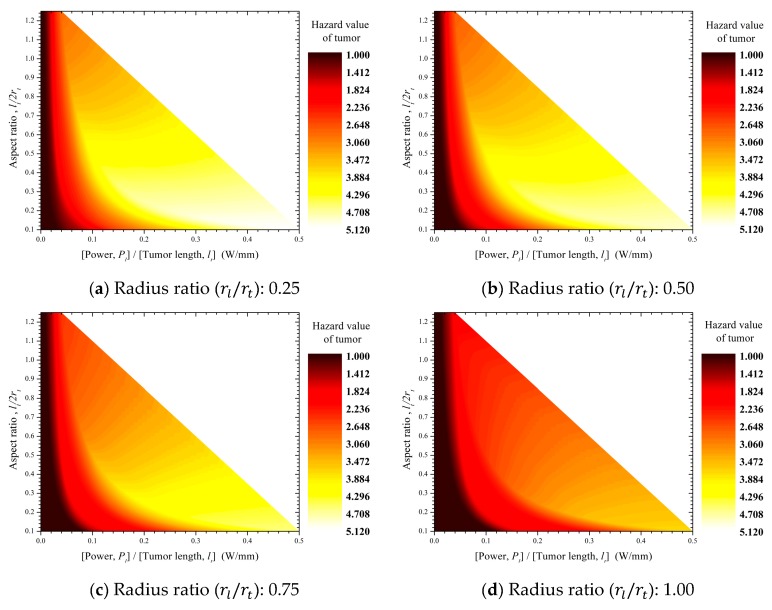

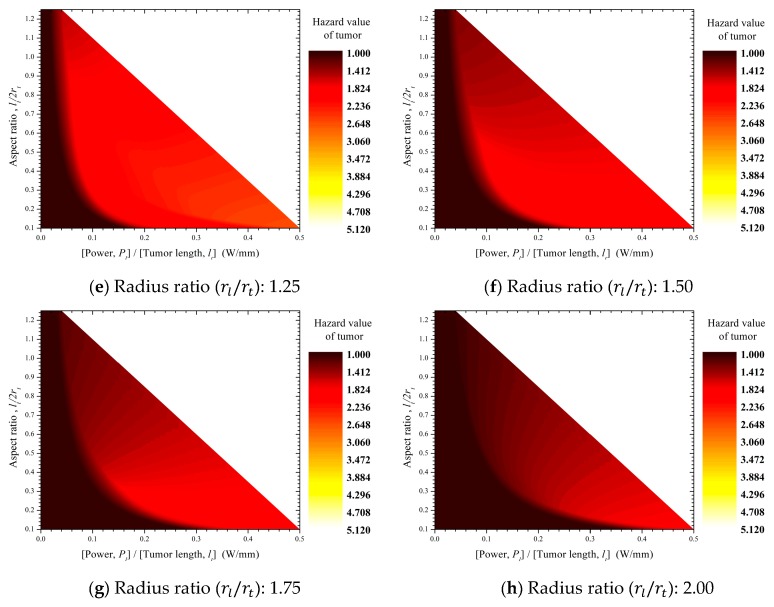
Contour graphs of the thermal hazard values of tumor tissues ($\theta_{H,t}$).

This apoptosis ratio of the tumor tissue can be explained using the thermally induced hazard value ($\theta_{H}$). Figure 10 shows the thermal hazard value ($\theta_{H,t}$) of the tumor tissue. The thermal hazard value ($\theta_{H,t}$) of the tumor increased as the tumor aspect ratio ($l_{t}/2r_{t}$) decreased and as the laser power per unit tumor length ($P_{l}/l_{t}$) increased. As shown in Figure 9, when the laser–tumor radius ratio ($r_{l}/r_{t}$) increases, uniform heating is induced by a change in the laser profile. When a laser with high power induces uniform heating, the temperature of the whole tumor exceeds the temperature range required for apoptosis.

In contrast, the thermal hazard value of the tumor (Figure 10) is very low ($\theta_{H,t}=1$) when the laser power per unit tumor length ($P_{l}/l_{t}$) is also in the low range. This result indicates that the temperature distribution of the tumor is $37\leq T_{t}<50$ when the weighted value of the hazard of tumor tissues in Table 1 is considered; thus, it cannot be concluded that the tumor has entered the apoptosis temperature range. This concept is also illustrated in Figure 8, which shows a very low apoptosis ratio in the low range of laser power per unit tumor length ($P_{l}/l_{t}$). This means that the tumor tissues were not sufficiently heated. In summary, the thermal hazard value is very low ($\theta_{H,t}=1$) in the low range of laser power per unit tumor length ($P_{l}/l_{t}$), which is due to insufficient heating of tumor tissues.

When we examined the apoptosis ratio ($\theta_{A}$) and thermal hazard value ($\theta_{H,t}$) of tumor tissues in the range where the apoptosis ratio of the tumor tissue is approximately 0.8 or higher, the thermal hazard value ranged from 1 to 1.5. This suggests that in the process of inducing apoptosis in tumor tissue, a small amount of necrosis may also occur when the temperature exceeds the range required for apoptosis.

### 3.2. Thermal Hazard Value of Normal Tissue

**Figure 11:**
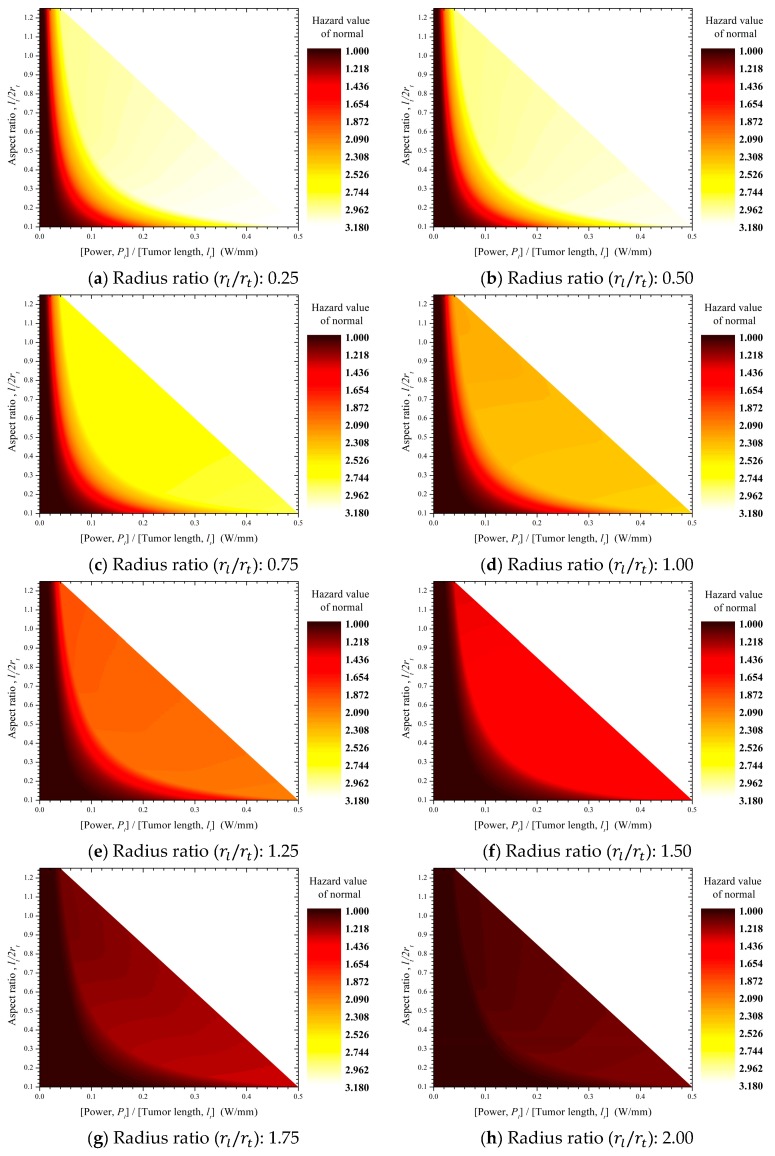
Contour graphs showing the thermal hazard values of normal tissues ($\theta_{H,n}$).

Figure 11 shows the thermal hazard values ($\theta_{H,n}$) of normal tissues according to the conditions of photothermal therapy. Regardless of the laser–tumor radius ratio ($r_{l}/r_{t}$), the thermal hazard values ($\theta_{H,n}$) of normal tissues show a trend similar to that of tumor tissues. Normal tissues have a low absorption coefficient ($\mu_{a}$) and therefore show almost no effects from photothermal heating. However, normal tissues still show an increased temperature, which is induced by thermal conduction from tumor tissues and can result in thermal damage.

An analysis of the apoptosis ratio ($\theta_{A}$, Figure 8) in tumor tissues revealed that the hazard value of normal tissue was approximately in the range of 1–1.3 for the tumor aspect ratio ($l_{t}/2r_{t}$) and laser power per unit tumor length ($P_{l}/l_{t}$) at which a high apoptosis ratio ($\theta_{A}$) was induced. A hazard value greater than 1 for normal tissues indicates that some regions of normal tissues exceed the safe temperature range (37≤T<43). This means that some normal tissues inevitably underwent thermal damage in the process of inducing a high apoptosis ratio ($\theta_{A}$) in tumor tissues.

### 3.3. Apoptosis Ratio of Tumor Tissue Evaluated with Respect to the Thermal Hazard Value of Normal Tissue

The purpose of photothermal therapy is to induce maximum apoptosis in tumor tissues while minimizing thermal damage to normal tissues. Therefore, in addition to evaluating the apoptosis ratio ($\theta_{A}$) in tumor tissues, it is important to consider the thermal hazard value ($\theta_{H,n}$) of normal tissues. For this, the effective apoptosis ratio in tumor tissues ($\theta_{A,eff}$) was defined with respect to the thermal hazard value of normal tissues as follows:

(10) $$\theta_{A,eff}=\frac{Apoptosisratio\;of\;tumor\;tissue(\theta_{A})}{Thermal\;hazard\;value\;of\;normal\;tissue(\theta_{H,n})}.$$

As shown above, a smaller thermal hazard value of normal tissues (range of $\theta_{H,n}$: 1–8) indicates a decreased risk for thermal damage to those tissues, while a larger apoptosis ratio ($\mathrm{range}\;\mathrm{of}\;\theta_{A}$: 0–1) indicates increased effectiveness of photothermal therapy. Therefore, it is important to achieve an effective apoptosis ratio in tumor tissues (range of $\theta_{A,eff}$: 0–1) while ensuring that the thermal hazard value of normal tissues remains close to 1.

**Figure 12:**
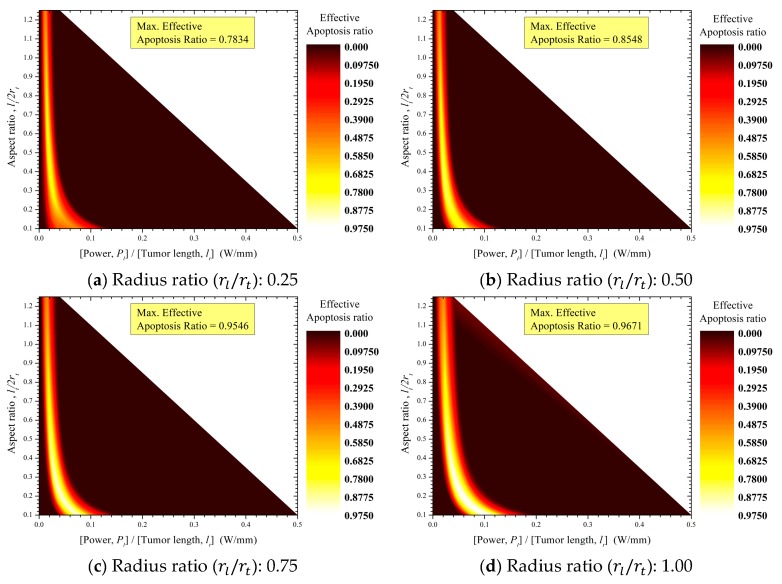

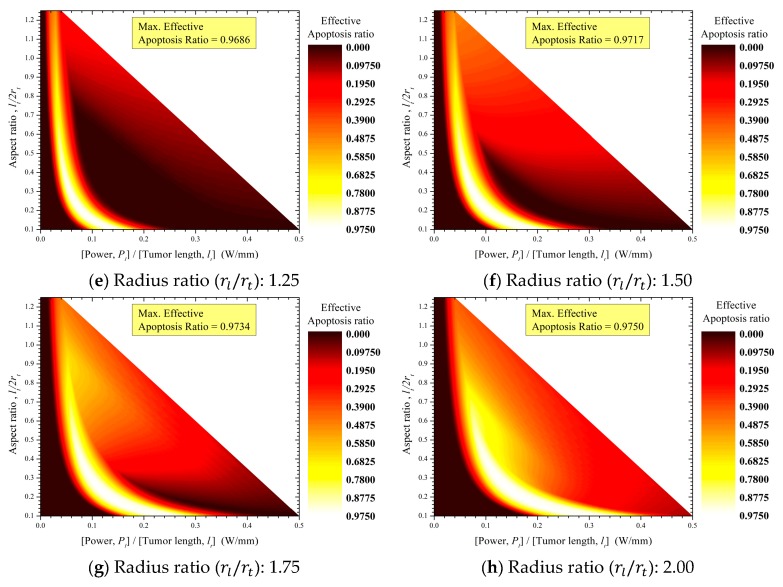
Contour graphs of the apoptosis ratios in tumor tissues with respect to the thermal hazard values of normal tissues.

Figure 12 shows the effective apoptosis ratios in tumor tissues ($\theta_{A,eff}$) determined using the tumor aspect ratio ($l_{t}/2r_{t}$) and laser power per unit tumor length ($P_{l}/l_{t}$) for various laser–tumor radius ratios ($r_{l}/r_{t}$). These results showed similar trends to the apoptosis ratios in tumor tissues ($\theta_{A}$, Figure 8). The maximally effective apoptosis ratio in tumor tissues converged to 0.975 as the laser–tumor radius ratio ($r_{l}/r_{t}$) increased. The reason for the similar tendency is that the thermal damage of normal tissues is also small at the condition wherein the effective apoptosis ratio of tumor tissues appears during photothermal therapy. In other words, the temperature range in which apoptosis occurs is 43‒50 °C, and the heat transferred at this temperature is too low to induce high levels of thermal damage. However, as shown in Figure 8; Figure 11, the thermal hazard value of normal tissues ranges from 1 to 1.3, while the apoptosis ratio in tumor tissues is approximately 0.8 or higher. Therefore, it is important to select effective treatment conditions to maximize the effective apoptosis ratio, as shown in Figure 12.

It should be noted that the effective conditions of photothermal therapy determined through these results of the effective apoptosis ratio are theoretical results. Therefore, optimal treatment using photothermal therapy should be clinically considered based on various biophysical phenomena.

## 4. Conclusions

In this study, we quantitatively derived the tumor apoptosis ratio ($\theta_{A}$) and thermal hazard value ($\theta_{H}$) of tumors and the surrounding normal tissues for tumors having various aspect ratios ($l_{t}/2r_{t}$) targeted using photothermal therapy via CW irradiation in the NIR region. The apoptosis ratio ($\theta_{A}$) was defined based on the apoptosis temperature criterion of 43–50 °C. The thermal hazard value ($\theta_{H}$) was defined by representing thermal damage as a weighted sum according to the biological effect of each temperature range. The effective apoptosis ratio ($\theta_{A,eff}$) was defined for tumor tissues in terms of the apoptosis ratio of tumor tissues ($\theta_{A}$) and the thermal hazard value of normal tissues ($\theta_{H,n}$). The results of our analysis were obtained using a numerical analysis model. The obtained apoptosis ratio ($\theta_{A}$) and thermal hazard value ($\theta_{H}$), derived using the parameters of tumor aspect ratio ($l_{t}/2r_{t}$), laser intensity ($P_{l}$), and laser radius ($r_{l}$), were generalized via nondimensionalization of the heat transfer equation. 

Based on the laser–tumor radius ratio ($r_{l}/r_{t}$), we found a range of tumor aspect ratios ($l_{t}/2r_{t}$) and laser powers per unit tumor length ($P_{l}/l_{t}$) in which the maximum apoptosis ratio ($\theta_{A}$) appeared. The thermal hazard values of tumor tissues ($\theta_{H,t}$) ranged from 1 to 1.5 in the range where the apoptosis ratio ($\theta_{A}$) of tumor tissues was approximately 0.8 or higher. This suggests that hazard effects in tumor tissues may also occur during the process of inducing apoptosis in tumor tissues. 

Thermal damage to normal tissues was predominantly affected by the temperature of the tumor tissues. A high apoptosis ratio ($\theta_{A}$) was observed when the hazard value was in the range of 1‒1.3 for tumor aspect ratio ($l_{t}/2r_{t}$) and laser power per unit tumor length ($P_{l}/l_{t}$). This suggests that induction of a high apoptosis ratio in tumor tissues ($\theta_{A}$) is inevitably accompanied by some thermal damage to normal tissues.

An effective apoptosis ratio ($\theta_{A,eff}$) can be defined in terms of the thermal hazard value of normal tissues ($\theta_{H,n}$) and apoptosis ratio of tumor tissues ($\theta_{A}$). Optimal determination of the tumor aspect ratio ($l_{t}/2r_{t}$) and laser power ($P_{l}$) is necessary for safe and effective treatment via photothermal therapy.

Further clinical trials will show whether it is more advantageous to induce maximal apoptosis in tumor tissues despite thermal damage to normal tissues or whether it is better to minimize the thermal damage to normal tissues even if the apoptosis ratio in tumor tissues is not 100% ($\theta_{A}=1$).

In this study, we applied a simplified model to perform the first step of quantitative analyses of photothermal therapy and determined laser conditions (power, $P_{l}$ and radius, $\;r_{l}$) with respect to tumor size (aspect ratio, $l_{t}/2r_{t}$), the apoptosis ratio ($\theta_{A}$) of the tumor, and the thermal hazard value ($\theta_{H}$) of tissues. However, this result is a numerical result from a simplified model, and further step-by-step studies and quantitative studies of models with complex and inhomogeneous characteristics similar to those of actual tumors are needed. The induction of apoptotic temperature in photothermal therapy is a very important factor, and the apoptosis ratio results of this study will help to select effective conditions to induce apoptosis of tumors. Photothermal therapy requires optimization for various treatment conditions or parameters. This study investigated effective treatment using photothermal therapy with an NIR CW laser in terms of apoptotic temperature and thermal damage to the surrounding normal tissue. However, an optimal treatment using photothermal therapy can be accomplished when both clinical and theoretical studies of photothermal therapy are considered. Through further research, optimal treatment with photothermal therapy should be established.

## Figures and Tables

**Table 1:** Laser-induced thermal effects [41].

Temperature Range (°C)	Biological Effect	Weight, $w_{j,t}$ (in Tumor Tissue)	Weight, $w_{j,n}$ (in Normal Tissue)
37≤T<43	Biostimulation	1	1
43≤T<50	Hyperthermia and reduction in enzyme activity	1	2
50≤T<70	Protein denaturation (coagulation)	2	3
70≤T<80	Welding	3	4
80≤T<100	Permeabilization of cell membranes	4	5
100≤T<150	Vaporization	5	6
150≤T<300	Carbonization	6	7
T>300	Rapid cutting and ablation	7	8

**Table 2:** Properties for verification of the numerical analysis.

Properties		Value
Normal tissue	Absorption coefficient ($\mu_{a,n}$, 1/m)	2
	Reduced scattering coefficient ($\mu_{s,n}^{\prime }$, 1/m)	650
	Density ($\;\rho_{n}$, kg/m^3^)	1000
	Specific heat ($c_{p,n}$, J/(kg∙K))	4200
	Thermal conductivity ($\;k_{n}$, W/(m∙K))	0.5
	Blood perfusion ($w_{b,n}$, s^−1^)	1.0 × 10^−3^
Tumor tissue with GNPs	Absorption coefficient ($\mu_{a,t}$, 1/m)	12,100
	Reduced scattering coefficient ($\mu_{s,t}^{\prime }$, 1/m)	50
	Density ($\;\rho_{t}$, kg/m^3^)	1100
	Specific heat ($c_{p,t}$, J/(kg∙K))	4200
	Thermal conductivity ($\;k_{t}$, W/(m∙K))	0.55
	Blood perfusion ($w_{b,t}$, s^−1^)	9.1 × 10^−4^
Other properties	Blood density ($\;\rho_{b}$, kg/m^3^)	1000
	Blood specific heat ($c_{p,b}$, J/(kg∙K))	4200
	Metabolic heat (${\dot{q}}_{mt},$ W/m^3^)	1091

**Table 3:** Properties evaluated in the numerical analysis.

**Optical Properties of GNPs (GNRs, Gold Nanorods) [37]**			
Aspect ratio	8.74	Absorption efficiency ($Q_{a,np}$)	50.326
Effective radius ($r_{np}$, nm)	3.9	Reduced scattering efficiency ($Q{’}_{s,np}$)	1.663
Maximum efficiency wavelength (λ, nm)	788	Extinction efficiency ($Q_{ext,np}$)	51.989
**Optical Properties of Skin [32]**			
Skin Type		Caucasian	
Absorption coefficient ($\mu_{a,m}$, 1/m)		0.115	
Reduced scattering coefficient ($\mu_{s,m}^{\prime }$, 1/m)		2409.7	
Extinction coefficient ($\mu_{ext,m}$, 1/m)		2409.8	
Wavelength ( λ, nm)		788	
**Thermal Properties [49]**			
		Tumor Tissue	Normal Tissue
Conductivity ( k, W/(m∙K))		0.5	0.0293
Density ( ρ, kg/m^3^)		1100	1000
Specific heat ($c_{p}$, J/(kg∙K))		4200	4200
Perfusion ($w_{b}$, s^−1^)		0.00091	0.001

**Table 4:** Parameters used for numerical analysis.

Numerical Parameter	Case	Number	Remarks
Fraction volume ($f_{v}$)	10^−5^	1	
Tumor radius ($r_{t}$)	2 mm	1	
Tumor length ($l_{t}$)	0.4 to 5 mm (intv: 0.2 mm)	24	Changed for the aspect ratio
Radius of the laser ($r_{l}$)	0.5 to 4 mm (intv: 0.5 mm)	8	
Power of the laser ($P_{l}$)	0 to 0.2 W (intv: 0.005 W)	41	
